# AHR2 required for normal behavioral responses and proper development of the skeletal and reproductive systems in zebrafish

**DOI:** 10.1371/journal.pone.0193484

**Published:** 2018-03-01

**Authors:** Gloria R. Garcia, Sean M. Bugel, Lisa Truong, Sean Spagnoli, Robert L. Tanguay

**Affiliations:** 1 Department of Environmental & Molecular Toxicology, Environmental Health Sciences Center, Sinnhuber Aquatic Research Laboratory, Oregon State University, Corvallis, OR, United States of America; 2 College of Veterinary Medicine, Oregon State University, Corvallis, OR, United States of America; Academia Sinica, TAIWAN

## Abstract

The aryl hydrocarbon receptor (AHR) is a conserved ligand-activated transcription factor required for proper vertebrate development and homeostasis. The inappropriate activation of AHR by ubiquitous pollutants can lead to adverse effects on wildlife and human health. The zebrafish is a powerful model system that provides a vertebrate data stream that anchors hypothesis at the genetic and cellular levels to observations at the morphological and behavioral level, in a high-throughput format. In order to investigate the endogenous functions of AHR, we generated an AHR2 (homolog of human AHR)-null zebrafish line (*ahr2*^osu1^) using the clustered, regulatory interspaced, short palindromic repeats (CRISPR)-Cas9 precision genome editing method. In zebrafish, 2,3,7,8-tetrachlorodibenzo-p-dioxin (TCDD) mediated toxicity requires AHR2. The AHR2-null line was resistant to TCDD-induced toxicity, indicating the line can be used to investigate the biological and toxicological functions of AHR2. The AHR2-null zebrafish exhibited decreased survival and fecundity compared to the wild type line. At 36 weeks, histological evaluations of the AHR2-null ovaries revealed a reduction of mature follicles when compared to wild type ovaries, suggesting AHR2 regulates follicle growth in zebrafish. AHR2-null adults had malformed cranial skeletal bones and severely damaged fins. Our data suggests AHR2 regulates some aspect(s) of neuromuscular and/or sensory system development, with impaired behavioral responses observed in larval and adult AHR2-null zebrafish. This study increases our understanding of the endogenous functions of AHR, which may help foster a better understanding of the target organs and molecular mechanisms involved in AHR-mediated toxicities.

## Introduction

The aryl hydrocarbon receptor (AHR) is highly conserved across multiple animal phyla and is present throughout metazoans [1]. AHR is a ligand-activated member of the basic helix-loop-helix PER-ARNT-SIM (bHLH/PAS) protein family of transcription factors [2, 3]. AHR was originally identified for its role in xenobiotic metabolism and severe toxicity of chlorinated dibenzo-*p*-dioxins and related compounds [4]. Many investigators use the receptor and its high-affinity ligand 2,3,7,8-tetrachlorodibenzo-*p*-dioxin (TCDD) as a molecular probe to gain insight into key biological processes, as well as the mechanism of AHR activation [5, 6]. Unliganded AHR is located in the cytosol and bound to a molecular complex that includes two molecules of Hsp90, XAP2, and p23. Upon ligand binding, the receptor undergoes a conformational change and is translocated to the nucleus, where it heterodimerizes with the class II bHLH protein ARNT (aryl hydrocarbon receptor nuclear translocator) or HIF1β (hypoxia-inducible factor 1β). The ligand-AHR-Arnt complex regulates transcription via binding to AHR responsive elements (AHREs) located in the promoter of target genes (e.g. Cyp1a1, a cytochrome P450 gene). The DNA-bound AHR-Arnt heterodimer is then disassembled, and AHR is transported to the cytosol for proteasomal degradation [7]. Additional mechanisms of AHR-dependent gene regulation have been recently identified, such as ligand-activated AHR forming heterodimers with nuclear proteins (e.g. RelB and Krüppel-like factor 6) that recognize DNA binding sites that are different from AHREs [8, 9]. Ligand-activated AHR can also act as a coactivator and form a complex with the transcription factor E2F1, which is important for transition of the cell cycle from G1 to S phase [10].

A diverse group of environmental pollutants can activate the AHR signaling pathway, including chlorinated dioxins, biphenyls, and polycyclic aromatic hydrocarbons. The inappropriate activation of the AHR can lead to adverse developmental and cognitive effects in wildlife and humans [11, 12]. Zebrafish have three isoforms of AHR (AHR1a, AHR1b, and AHR2) due to an ancient genome duplication event that resulted in co-orthologs for a large number of mammalian genes [1, 13]. In zebrafish, AHR2 and ARNT1 are the primary functional orthologs of mammalian AHR and ARNT that mediate early life stage toxicological effects [1, 12, 14]. Due to substitutions in critical amino acid residues in the ligand-binding domain of AHR2, the zebrafish is among the most sensitive vertebrate models making it optimal for investigating AHR signal transduction and function [15, 16]. The toxic responses between mammalian and zebrafish models are very similar because of their well-conserved genomes, cell types, tissues, and organ systems [17, 18].

The AHR regulates multiple functional processes beyond toxicology. In both mice and zebrafish, the absence of a functional AHR protein results in reduced fecundity, a slower growth rate, and lower survival rates in offspring [19, 20]. In AHR-null neonatal mice, necropsy revealed lymphocyte infiltration in the lung, gut, and urinary tract suggesting the cause of death was related to an opportunistic infection. Upon further examination of the immune system, AHR-null mice had a delay in splenic lymphocyte development and an overall reduction of lymphocytes in the lymph nodes and spleen [19, 21]. AHR-mutant mice also displayed impaired liver function with pronounced fibrosis in the portal tract visible by 3 weeks post birth. AHR2-null zebrafish had noticeable neurocranial aberrations due to abnormally structured bones of the head and severely damaged dorsal, ventral, and caudal fins in adults [20]. In summary, the AHR is required for proper vertebrate organ development, immune responses, and reproductive function.

The previously characterized AHR2 zebrafish mutant line (*ahr2*^hu3335^) was generated using the targeted induced local lesions in genomes (TILLING) method, that was developed to randomly knock out gene function in ENU-mutagenized zebrafish [22]. A major drawback to this method is the requirement for multiple generations of outcrossing due to the high degree of background mutations. This leads to some uncertainty whether the observed phenotypes described from Goodale *et al*. 2012 were solely due to the single nucleotide AHR2 mutation. Recent advancements in precision genome editing in zebrafish include the use of the clustered, regulatory interspaced, short palindromic repeats (CRISPR)-Cas9 system, which relies on a single guide RNA (sgRNA) and the Cas9 nuclease to generate targeted double strand breaks next to specific recognition sites called protospacer adjacent motifs [23]. In this study, we used the CRISPR-Cas9 method to generate and characterize an AHR2-null zebrafish line (*ahr2*^osu1^) to investigate the endogenous functional role of AHR2 in zebrafish behavioral responses, organ development, and reproduction.

## Materials and methods

All gene-specific primers and sgRNAs are listed in S1 Table.

### Fish husbandry

The Tropical 5D line (*ahr2*^+^) of zebrafish (*Danio rerio*) and *ahr2*^osu1^ mutants were reared according to Institutional Animal Care and Use Committee (IACUC) protocols at the Sinnhuber Aquatic Research Laboratory, Oregon State University. The approval number associated with this manuscript is 4748. Adult animals were raised in a recirculating water system (28 ± 1°C) with a 14-h:10-h light-dark schedule. Adult wild-type fish were kept at a density of 6–8 fish per liter. Adult *ahr2*^osu1^ fish were kept at a lower density of 4 fish per liter because of their fin malformations. Spawning and embryo collection were conducted as described in [24]. Adult and larval zebrafish were euthanized with Tricane (3-amino benzoic acid ethylester) at concentrations exceeding 300–400 mg/L and 150–200 mg/L, respectively.

### Design, assembly, and preparation of sgRNA

The https://chopchop.rc.fas.harvard.edu/ website was used to generate two AHR2 single guide RNAs (sgRNAs) to target exon1 and for sgRNA off-target analysis as described in [25]. An updated and improved version of the website is listed at http://chopchop.cbu.uib.no/. We used the cloning-free method described in [26] with the following modification to assemble the sgRNAs. The sgRNA templates were generated using a gene-specific oligo containing the Sp6 promoter, an 18–20 nt target sequence, and a 23 nt sequence that overlapped a constant (generic) oligo sgRNA template. The fill-in method using T4 DNA polymerase did not produce specific bands, so we designed universal forward and reverse primers that matched the 20 nt sequence at the 5’ end of the gene-specific and 3’ end of the constant oligos, respectively. The template was amplified using KOD Hot Start DNA Polymerase (Novagen; Japan) with 0.2 μM each of the gene-specific oligo, forward, and reverse primer, and 0.008 μM of the constant oligo under the following conditions: denaturation at 95 °C for 2 min, followed by 35 cycles of amplification (95 °C for 20 sec, 62 °C for 10 sec, 72 °C for 10 sec), and a final extension at 72 °C for 5 min. The DNA was precipitated using ethanol/sodium acetate (pH 5.2). The quality of the DNA template was checked on a 1.2% agarose gel. The DNA was quantified using a SynergyMix microplate reader with the Gen5 Take3 module. Approximately 2 μg of DNA template was used to transcribe the RNA using the Sp6 RNA Polymerase in vitro transcription components (Promega; Madison. WI) according to the manufactures instructions. The sgRNAs were precipitated using ethanol/ammonium acetate (pH 5.2). The quality of the sgRNAs was checked on a 1.2% agarose gel. The quantity was assessed using a SynergyMix microplate reader with the Gen5 Take3 module.

### Embryo injections

The microinjections were performed in the Tropical 5D wild-type strain. We injected a sgRNA/Cas9 nuclease solution for each individual sgRNA. Approximately 300 pg of a sgRNA and 175 pg of purified Cas9 protein with a nuclear localization signal derived from SV40 Large T Ag (Clonetech; Mountain View, CA) were injected into the cell of a one-celled embryo.

### Founder screening

Injected fish were raised to 2–4 months of age and screened for germline transmission of CRISPR-induced mutations by applying fluorescence PCR followed by melt curve analysis (described below). Each potential founder (mutant to establish the line) was crossed with a 5D wild-type fish and eight 4–5 dpf embryos from each pair spawn (1 founder and 1 wild type) were plated into a 96-well plate, with one embryo per well. Eleven potential founders and eight wild-type controls were screened/plated. For DNA extraction, embryos (4–5 dpf) were euthanized using ice, and excess water was removed from the wells. Embryos were lysed by incubated in 20 μl of 50 mM NaOH at 95°C for 10 minutes and vortexed for 10–15 seconds. The plate was allowed to cool for 5 minutes at room temperature and mixed with 4 μl of 1 M Tris-HCl (pH 8.0) neutralization solution by vortexing. The DNA was diluted with 80 μl of ultrapure water to reduce PCR inhibitors and spun down at 4680 rpm for 10 minutes to pellet debris to the bottom of the plate.

### Fluorescence PCR and melt curve analysis

The 20 μl PCR reactions consisted of 10 μl 2X SYBR Green Master Mix (Applied Biosystems; Foster City, CA), 0.4 μl each of 10 μM forward and reverse primers, and 1.5–2 μl of diluted DNA. The primers were designed to be 10–15 bp away from the predicted Cas9 cut site (-3 nt from PAM site) and produce 60 to 70 nt products. The reaction was completed using a StepOnePlus Real-Time PCR System (Applied Biosystems; Foster City, CA) under the following conditions: denaturation and activation of SYBR polymerase at 95 °C for 5 min, followed by 40 cycles of amplification (95 °C for 15 sec, 58 °C for 1 min), followed by melt curve analysis with a ramp rate of +0.2 (95 °C for 2 min, 70 °C for 2 min, 95 °C for 2 min). Founders were identified by a shift in the melt-curve peak relative to the wild type control. Using this method, we were able to detect mutations as small as 2 nt. To determine if the identified founders produce frameshifting mutations, we amplified a region 200–400 nt up- and downstream of the sgRNA target site in a 25 μl PCR reaction that had a 0.2 μM final concentration of forward and reverse primers, 2 μl of diluted DNA, and KOD Hot Start DNA Polymerase (Novagen; Japan) PCR reaction components according to manufactures instructions. The PCR conditions were as follows: denaturation at 95 °C for 2 min, 35 cycles of amplification (95 °C for 20 sec, 62 °C for 10 sec, 72 °C for 10 sec), followed by an extension at 72 °C for 5 min. Sanger sequencing was performed by the Core Facilities of the Center for Genome Research and Biocomputing at Oregon State University using an ABI 3730 capillary sequence machine. Sequencing results for mixed base reads in heterozygous founders at the mutation site were interpreted manually or using various online tools for characterizing indel mutations (https://tide-calculator.nki.nl/ and http://yosttools.genetics.utah.edu/PolyPeakParser/).

### Waterborne exposure

Shield-stage (~6 hpf) embryos were exposed to 1 ng/mL TCDD in embryo medium (311 nM, 95.3% purity; SUPELCO Solutions Within; Bellfonte, PA; CAS number 1746-01-6) or vehicle (0.1% DMSO in embryo medium) with gentle rocking for 1 hour in 20 mL glass vials (10 embryos/mL). Vials were also gently inverted every 15 minutes to ensure proper mixing. After the exposure, embryos were rinsed 3 times with embryo medium and then raised in 100 mm petri dishes.

### Adult microCT scan

Micro computed tomography (microCT) was used to generate three-dimensional imaging of ~8 month old adult zebrafish heads. Two male and two female adult zebrafish were scanned for each genotype. The fish were scanned using a Scanco μCT40 scanner (Scanco Medical AG, Basserdorf, Switzerland) at 45 kVp, 177 mA, and a voxel size of 12×12×12 mm (threshold = 140).

### Cartilage staining and lower jaw cartilage morphometrics

Zebrafish embryos were microinjected with control and *slincR* MOs and exposed to 0.1% DMSO, or 1 ng/mL TCDD as described above. At 120 hpf, larvae were euthanized with Tricaine and fixed in 4% paraformaldehyde overnight at 4 °C. Pigmentation was removed by incubating fixed larval samples for 1 hour in a mixture of 3% H_2_O_2_/1% KOH. The cartilage was stained with 0.4% Alcian Blue 8GX (Sigma-Aldrich, St Louis, MO) in 70% ethanol and 80 mM MgCl_2_ as described in [27]. Larvae were imaged in 0.8% low-melt agarose at room temperature using a Keyence BZ-x700 at 10X with 0.45 aperture and processed with the BZ-x Analyzer software. Morphometric analysis (*n* = 9–10 embryos) of ventral larval pharyngeal cartilages was performed using ImageJ v1.51j8 and two customized macros (S1 and S2 Files) as described in [28]. Statistical significance was determined with the multcomp (version-1.4–6) and sandwich (version-2.4–0) packages in R using a modified one-way ANOVA and Tukey multiple comparison of means that is robust in regards to equal variance, sample sizes, and distribution [29–32], and data were graphed using ggplot2 [33].

### RNA extraction and mRNA quantification

Total RNA was extracted from 120 hpf whole embryos using RNAzol (Molecular Research Center, Inc; Cincinnati, OH) and a bullet blender with 0.5 mM zirconium oxide beads (Next Advance, Averill Park, New York) as recommended by the manufacturer. The RNA was purified using the Direct-zol MiniPrep kit (Zymo Research; Irvine, CA) and included an in-column DNase 1 digestion. RNA quality and quantity was measured using a SynergyMix microplate reader with the Gen5 Take3 module. Each biological sample consisted of 4 embryos with 4 biological replicates per condition. Total RNA (500 ng) was reverse transcribed into cDNA with random primers using the ABI High-Capacity cDNA Reverse Transcription Kit (Thermo Fisher; Eugene, OR). Quantitative real-time PCR (qRT-PCR) was performed using a StepOnePlus Real-Time PCR System (Applied Biosystems; Foster City, CA). The 20 μl reactions consisted of 10 μl 2X SYBR Green Master Mix (Applied Biosystems; Foster City, CA), 0.4 μl each of 10 μM forward and reverse primers, and 15 ng cDNA. Expression values were normalized to β-actin and analyzed with the 2-ΔΔCT method as described in [34]. The data for each gene were tested for normality using the Shapiro-Wilk normality test and for equal variance using the Levene’s test for homogeneity of variance. The data were statically analyzed in R using a two-sample t-test or Welch’s t-test for data that passed or failed normality and variance testing, respectively. The Holm-Sidak method for multiple comparisons was used with α = 0.05. For comparisons with more than two groups, a two-way ANOVA with correction for multiple comparisons was performed using the Dunnett’s test.

### Reproductive assessments: Fecundity and histopathology

Reproductively active adult zebrafish were age matched and pair spawned every two weeks to evaluate fecundity for wild type and AHR2-null mutants at 6–8 months post-fertilization. To condition animals for regular interval pair spawning, adult zebrafish were bred in large groups at 24 and 26 weeks post-fertilization (10 male with 10 females per group). At 28, 30, and 32 weeks post-fertiization, 5 small groups were spawned for each genotype, each including 2 males and 2 females. After each spawning event, each small group from each genotype was recombined and group housed so that each spawn attempt was a randomly selected group of animals. At 34 weeks post-fertilization, a group spawn was again performed (10 males with 10 females). Total embryos produced and viability at 120 hpf were recorded for each group. For group spawns (24, 26, and 34 weeks), a Fisher’s exact test was used to determine significance between genotypes. For small group spawns (28, 30, and 32 weeks) significance was determined using a two-way ANOVA with Tukey’s post hoc test for multiple comparisons (p < 0.05).Differential follicle analysis was performed using age-matched adult cohorts to determine representation of oocyte developmental stages. Two cohorts were evaluated, and the approximate ages of the younger and older cohorts were relatively 20 and 36 weeks post fertilization—corresponding to the early-mid stages of the zebrafish lifespan. All histopathological evaluations were performed blind. Adult whole animals were fixed and prepared for histology using Dietrich’s fixative with 5% trichloroacetic acid, and stored in 70% ethanol. Fixed animals were embedded in paraffin, sectioned (6 μm thickness) and stained with hematoxylin and eosin. For each animal, two para-sagittal sections were evaluated, each on opposite sides of the mid-sagittal plane (n = 4 animals per genotype and time point). For each section, the ovary was digitally captured using a Keyence BZ-X710 microscope and all follicles scored (typically >100 follicles per section). Ovarian follicles were identified using histopathological criteria previously described for the major stages of oocyte development, including Stage I (pre-vitellogenic primary growth), Stage II (early-vitellogenic cortical alveolus stage), Stage III (mid-vitellogenic), Stage IV (late-vitellogenic mature), and atretic follicles [35]. Prior to statistical analysis, a Box-Cox power transformation was applied to meet normality conditions. Statistical differences between genotypes (*ahr2*^*+*^ and ahr2^osu1^) and follicle stages within each genotype was determined using a two-way ANOVA with Tukey’s post hoc test for multiple comparisons (p < 0.05).

### Larval morphology

Zebrafish embryos (n = 32) were exposed to either embryo medium, 0.1% DMSO, or 1 ng/mL TCDD as described above and placed into 96-well plates after hatching (~48–60 hpf). Zebrafish were evaluated at 120 hpf for mortality and a suite of 17 physical malformations as described in [36]. The morphology data were analyzed using a Fisher’s exact test because it uses low category counts and does not make distributional assumptions (in contrast with the chi-squared test). The Bonferroni correction for multiple comparisons was used to control for family-wise error rate [37].

### Larval behavior assay

Zebrafish wild type and mutant embryos (n = 32) were placed into 96-well plates (100 uL embryo medium per well) after hatching (~48–60 hpf). The larval photomotor response (LPR) assay consisted of 3 min light and dark alternating periods, for a total of four light-dark transitions, with the first transition representing an acclimation period. The ViewPoint ZebraBox system (ViewPoint Behavior Technology) was used to analyze photo-induced larval locomotor activity in 120 hpf larvae. We followed the protocols and analyzed the overall area under the curve for the last three light-dark cycles compared to wild-type fish using a Kolmogorov-Smirnov test (p < 0.01) as described in [38]. We used a 1% alpha to control for type I error inflation rather than controlling the family-wise error rate as we are not correcting for multiple treatments, but only a wild type (*ahr2*^*+*^*)* and AHR2-null (*ahr2*^osu1^) group.

### Adult behavior assays

Adult zebrafish, 32 fish per wild-type or ahr2^osu1^ mutant zebrafish (4 groups of 8 adult zebrafish), ~ 8 months old, were tested over a 3 day period. The zebrafish visual imaging system (zVIS) was used to measure innate predator avoidance, social cohesion, and startle response as described in [39]. Briefly, the startle stimulus, a tap was generated by an electronic solenoid below each tank and the fish received a total of 5 taps with 20 second latency intervals, and total movement after each tap was measured. For the predator response and social cohesion assays, the fish were placed in a tank with single side views of LCD video projections displaying either a predator fish attacking and consuming its prey or shoaling zebrafish, respectively. The tank was subdivided into three zones in relation to the video projection (close, middle, and far) and we tracked the time the fish spent in each zone. For the predator test, zebrafish were expected to flee from the video projection. Statistical differences between mutants and controls were determined by two-way ANOVA with repeated measures and Tukey’s HSD post hoc test (p < 0.05).

## Results

### Generation of mutant line

**Fig 1:**
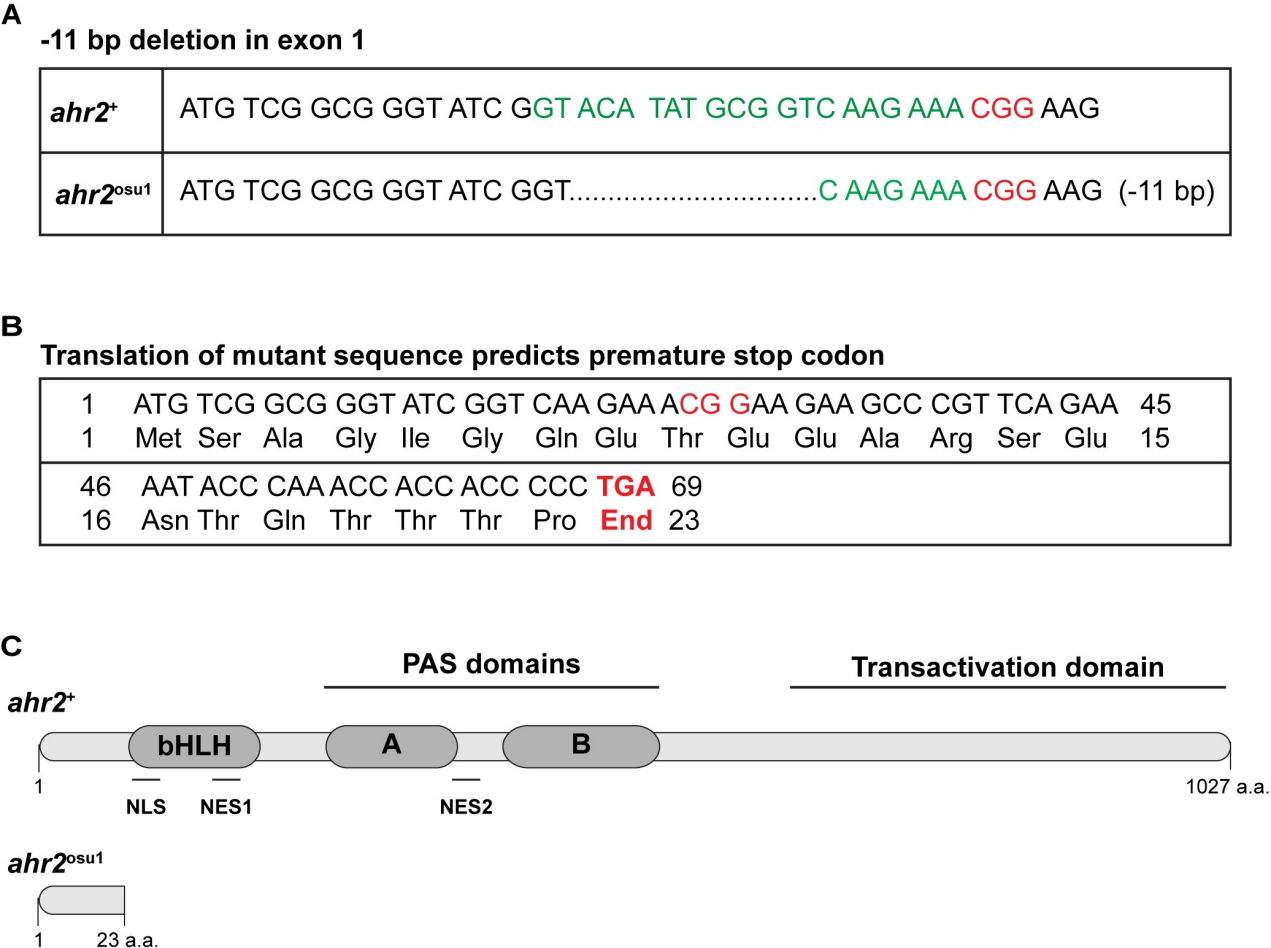
Schematic diagram of the mutation sequence and predicted AHR2 protein. **(A)** The AHR2 exon 1 DNA sequence in *ahr2*^+^ (top) and *ahr2*^osu1^ (bottom) zebrafish. The *ahr2*^osu1^ mutant has an 11 bp deletion. The target sequence is in green and the PAM site is in red (CGG). **(B)** The translated mutant sequence results in a frameshift mutation and is predicted to result in a premature stop codon at amino acid residue 23. **(C)** Schematic diagram of the *ahr2*^+^ (top) and predicted *ahr2*^+^ (bottom) protein. The predicted truncated protein does not contain the bHLH, PAS, or transactivation domains. NLS = nuclear localization signal, NES1 = nuclear export signal 1, NES2 = nuclear export signal 2.

We generated a zebrafish AHR2-null line (*ahr2*^osu1^) on the wild type 5D Tropical background using the CRISPR/Cas9 system. We designed two sgRNAs that targeted exon 1 of the AHR2 locus. We identified a founder with an 11 nt deletion that resulted in a premature stop codon upstream of the nuclear localization and export signals, and the bHLH, PAS, and transactivation domains (Fig 1). The predicted mutant protein should not be localized to the nucleus nor be capable of binding a ligand or DNA element, thus rendering the protein functionally null.

To generate homozygous *ahr2*^osu1^ mutants, heterozygous in-crosses were performed. We genotyped the putative mutant line during larval development and in adulthood. The genotype screen performed on 5 dpf larval zebrafish (n = 91, whole larvae) produced the approximate expected Mendelian genotype ratios for wild type *ahr2*^*+*^ (25.3%), heterozygous *ahr2*^osu1/+^ (48.4%), and homozygous *ahr2*^osu1^ mutants (26.4%). Genotyping fin clips of 2–3 month old zebrafish (n = 515 fin clips) produced genotype ratios of *ahr2*^*+*^ (33.8%), *ahr2*^osu/+^ (53.0%), and *ahr2*^osu1^ (13.2%), which suggested the AHR2-null zebrafish had reduced survival to adulthood.

### *ahr2*^osu1^ mutant is TCDD-resistant

**Fig 2:**
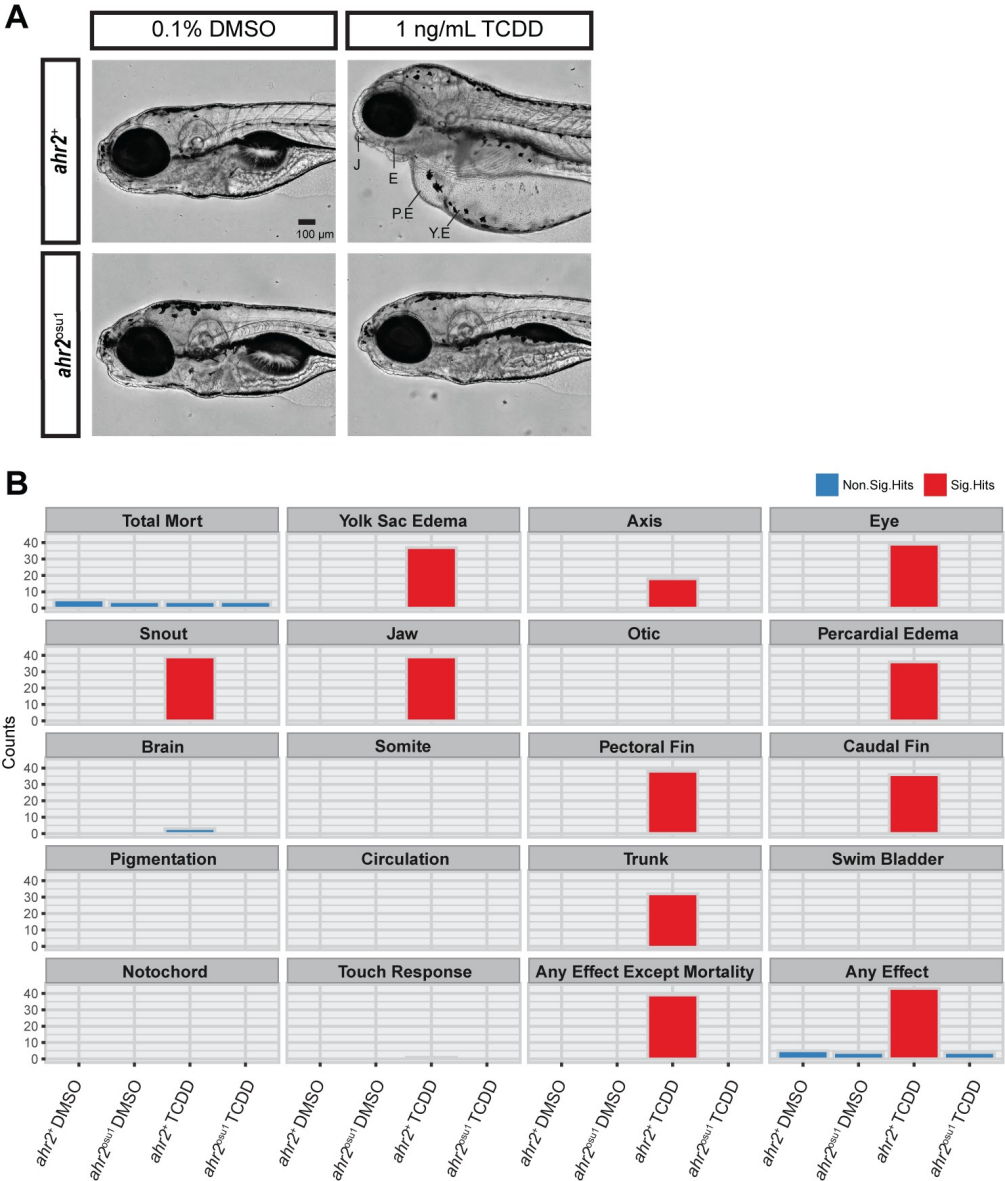
*ahr2*^osu1^ mutants are resistant to TCDD-induced developmental toxicity. **(A)** Lateral view of representative bright field images of 5 dpf *ahr2*^+^ and *ahr2*^osu1^ zebrafish developmentally exposed to embryo medium (EM), 0.1% DMSO, or 1 ng/mL TCDD. J = jaw, E = eye, PE = pericardial edema, and YE = yolk sac edema. Black bar in bottom right corner = 100 μm. **(B)** A 5 dpf zebrafish embryo-larval developmental toxicity assay for *ahr2*^+^ and *ahr2*^osu1^ mutants developmentally exposed to DMSO or TCDD (*n* = 32). The wild-type embryos exposed to TCDD exhibited significant malformations for 11 endpoints examined, including yolk sac and pericardial edema, and craniofacial malformations. No significant malformations were observed in the AHR2^-^null zebrafish exposed to TCDD. Morphological evaluations were completed in a binary notation (present/absent) and statistically compared using Fisher’s exact test at *p* < 0.05 for each endpoint.

AHR-knockout mice and zebrafish have previously shown that the AHR is required for TCDD-induced toxicity [19, 20]. To determine the functionality of the AHR2 protein in our mutant line, we developmentally exposed *ahr2*^osu1^ and *ahr2*^+^ zebrafish at 6 hpf to 0.1% DMSO or 1 ng/mL TCDD for 1 hour. We compared developmental toxicity using a 17 endpoint morphology screen at 120 hpf, as described in [36]. Developmental exposure to TCDD resulted in the expected malformation phenotype in the wild-type strain; however, the *ahr2*^osu1^ strain was resistant to TCDD-induced developmental toxicity demonstrating that the AHR2 protein in our CRISPR line was non-functional (Fig 2A and 2B). The wild-type strain exposed to TCDD had significant effects for the following morphological endpoints: yolk sac and pericardial edema, eye, jaw, snout, trunk, pectoral fin, and caudal fin (Fig 2A and 2B).

**Fig 3:**
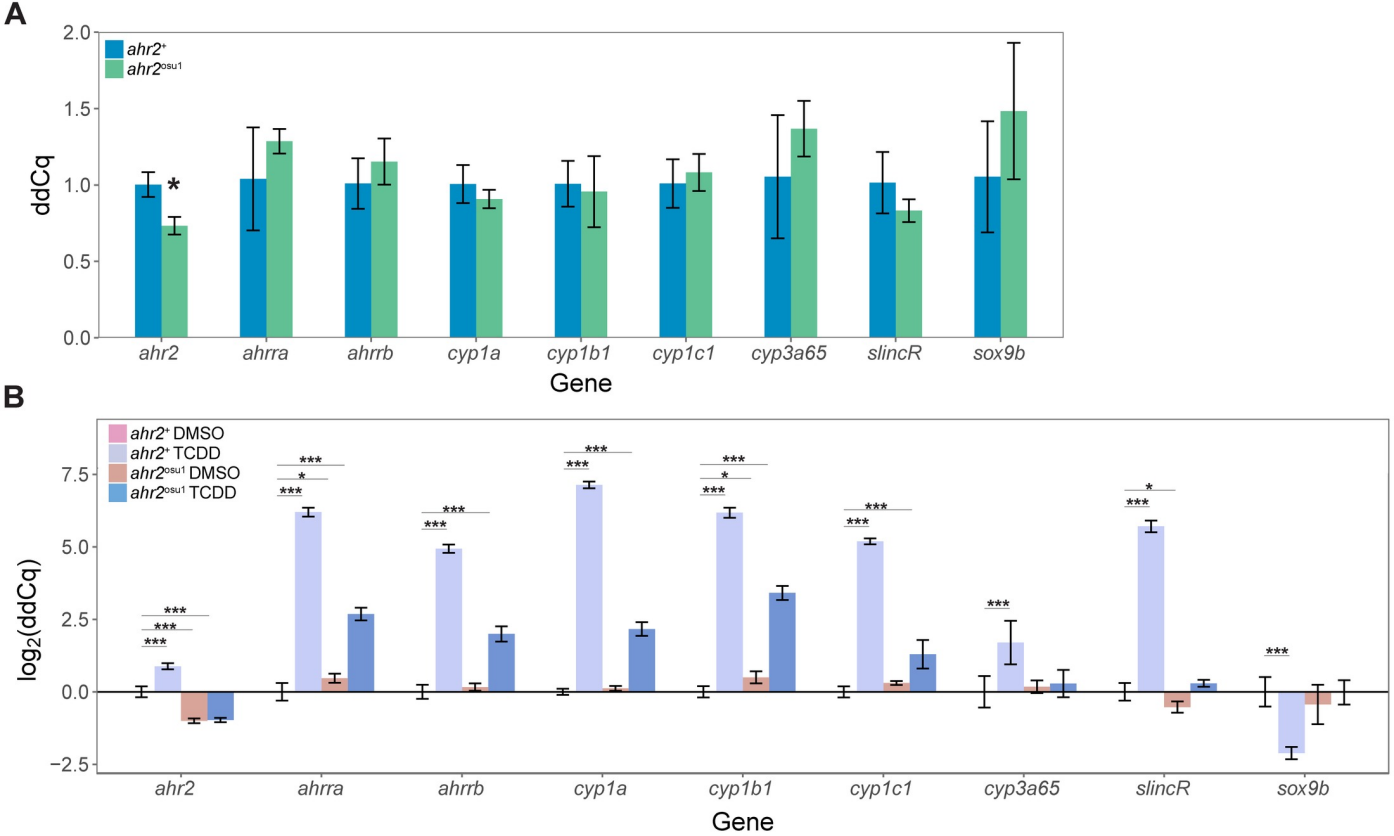
*ahr2*^osu1^ mutants exhibit reduced *ahr2* mRNA expression levels and reduced expression of known AHR2 target genes. **(A)** Comparative gene expression of *ahr2* and known transcripts downstream of AHR2 activation in *ahr2*^+^ and *ahr2*^osu1^ zebrafish at 5 dpf (*n* = 4 biological replicates with 4 embryos per replicate). Data for each gene was tested for normality using the Shapiro-Wilk normality test and equal variance using the Levene’s test for homogeneity of variance. Data was statistically analyzed in R using a two-sample *t*-test or Welch’s two-sample *t*-test for data that either passed or failed equal variance testing, respectively. The Holm- Šídák multiple comparisons method was used with *α* = 0.05. **(B)** Comparative gene expression of *ahr2* and known transcripts downstream of AHR2 activation in 5 dpf wild-type and *ahr2*^osu1^ zebrafish developmentally exposed to 0.1% DMSO or 1 ng/mL TCDD (*n* = 4 biological replicates with 4 embryos per replicate). Data for each transcript was tested for normality and equal variance as described above. Statistical significance was analyzed using a two-way ANOVA and a correction for multiple comparisons was performed using the Dunnett’s test. For all qPCR data, expression values were analyzed with the 2^-ΔΔCT^ Pfaffl method and normalized to β-actin. Error bars indicate SD of the mean. * = *p* < 0.05, ** = *p* < 0.01, and *** = *p* < 0.001 compared to either **(A)** *ahr2*^+^ or **(B)** *ahr2*^+^ vehicle control (DMSO).

To further assess the function of AHR2, we examined mRNA expression of *ahr2* and transcripts downstream of AHR2 activation in 120 hpf wild-type and mutant zebrafish. We observed a modest but significant decrease in *ahr2* expression in the *ahr2*^osu1^ strain, when compared to the *ahr2*^+^ line (**Fig 3A**). To determine the TCDD-induced gene expression changes, we exposed the *ahr2*^+^ and *ahr2*^osu1^ strains at 6 hpf to 0.1% DMSO or 1 ng/mL TCDD for 1 hour and collected total RNA at 120 hpf. In response to TCDD, both the *ahr2*^+^ and *ahr2*^osu1^ larval fish had a significant increase in the relative expression levels of *cyp1a*, *cyp1b1*, *cyp1c1*, *ahrra*, and *ahrrb*, in comparison to the vehicle-treated wild type; however, the wild type fish exposed to TCDD had a greater log_2_ fold increase in expression of known AHR2 target genes (Fig 3B). In zebrafish, developmental exposure to TCDD causes an AHR2-dependent increase in *cyp3a65* and *slincR* transcript levels and a decrease in *sox9b* mRNA expression [40, 41]. Only the wild-type strain resulted in a significant increase in *cyp3a65* and *slincR* expression and decrease in *sox9b* expression in response to TCDD exposure.

### Impaired reproduction/fertility in AHR2 mutants

**Fig 4:**
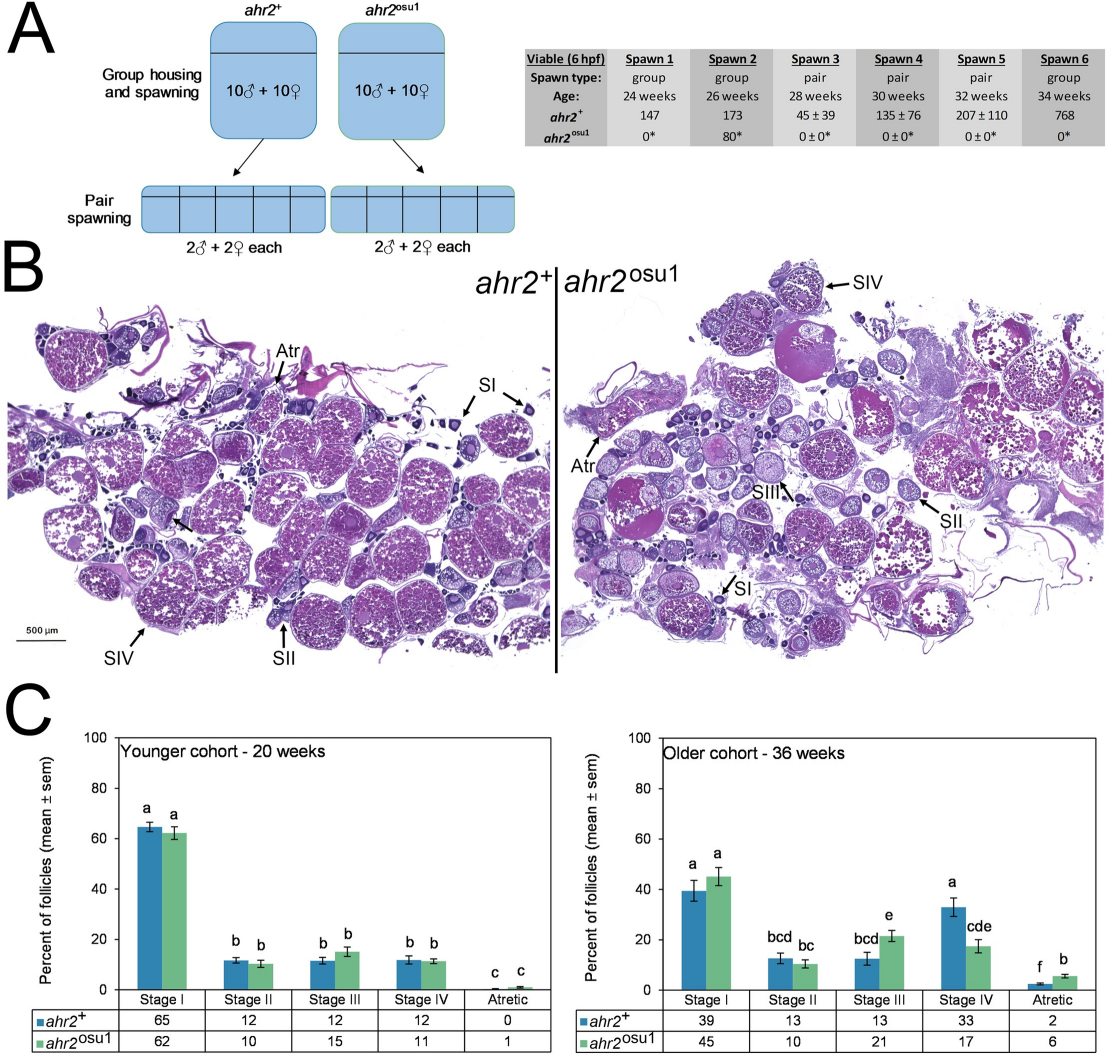
Reproductive impacts on the ovary are evident in *ahr2*^osu1^ zebrafish. **(A)** A fecundity study was performed with group and pair spawning between weeks 24 and 34 with age-matched cohorts of *ahr2*^+^ and *ahr2*^osu1^ zebrafish. Weeks 24–34 correspond to the optimal reproductive period. Between spawns, pairs were re-combined into groups so that each pair spawn were randomly selected groups. *p < 0.05 for group spawning (Fisher’s exact test) or pair-wise spawning (Student’s t-test) when compared to wild type. For group spawns, n = 10 males, 10 females. For pair spawns, n = 5, each with 2 males and 2 females. **(B)** Ovarian histopathological assessments were performed on *ahr2*^+^ and *ahr2*^osu1^ zebrafish to quantify the representative follicle distribution. Photomicrographs for the older cohort (36 weeks) are shown for each genotype using the sections most representative of group averages. **(C)** Differential follicle analysis was performed for reproductively active adult zebrafish at 20 and 36 weeks (n = 4 per genotype for each age group). Statistical differences between genotypes and developmental stages were determined using two-way ANOVA with Tukey *post hoc* test for multiple comparisons (*p* < 0.05). Significance is indicated using compact letter display, and bars not in the same letter group are significantly different. Follicles were scored as Stage I (pre-vitellogenic primary growth), Stage II (early-vitellogenic cortical alveolus stage), Stage III (mid-vitellogenic), Stage IV (late-vitellogenic mature), and atretic.

To evaluate AHR2-null zebrafish for effects on reproductive health, several endpoints were evaluated including fecundity and histopathology of the gonads. First, a fecundity study was performed between weeks 24 and 34, which demonstrated that AHR2-null zebrafish produced fewer eggs than wild type animals using various spawning parameters (Fig 4A). For these controlled studies, spawning was performed in groups or pairs. At weeks 24 and 26, group spawning was performed to condition previously unspawned animals to a 2 week spawn cycle. A group spawn at week 26 was the only spawning event to produce embryos, which were relatively fewer than wild type. Pair spawning performed at weeks 28, 30, and 32 produced no embryos by *ahr2*^osu1^ mutants, whereas wild type produced robust clutches of embryos. One last group spawn was attempted at week 34, which also produced no embryos from the AHR2-null group. The 20 and 36 week post-fertilization cohorts of animals were also processed for histopathological evaluations.

Whole animal histopathological evaluations for two age groups, including 36 week old animals that were the same as those used in the fecundity study, and a separate group of animals that were 20 weeks old were performed including all major tissues and structures, with few remarkable findings reported. With the exception of mild to severe egg associated inflammation in all four of the 36 week and two of the 20 week *ahr2*^osu1^ females, while only one out of the four 20 week wild type females exhibited mild egg associated inflammation. Histopathology of the female gonads was further evaluated to determine the developmental stages of ovarian follicles (Fig 4B). Stages were: Stage I (primary oocyte), stage II (early vitellogenic follicles with cortical alveoli), stage III (mid-vitellogenic follicle), stage IV (mature follicle), and atretic follicles (degenerative mature follicles). Within the younger cohort at week 20, no significant differences were found between genotypes (wild type and AHR2-null zebrafish) (Fig 4C). However, at 36 weeks post-fertilization, the *ahr2*^osu1^ genotype exhibited an increased percentage of Stage III (mid-vitellogenic) follicles, a decreased percentage of Stage IV (mature follicles), and an increase prevalence of atretic follicles (Fig 4C). In addition, these results suggest that the AHR2 is necessary for follicular development for normal female reproduction.

### Zebrafish AHR2 is required for normal adult fins and skeletal structures

**Fig 5:**
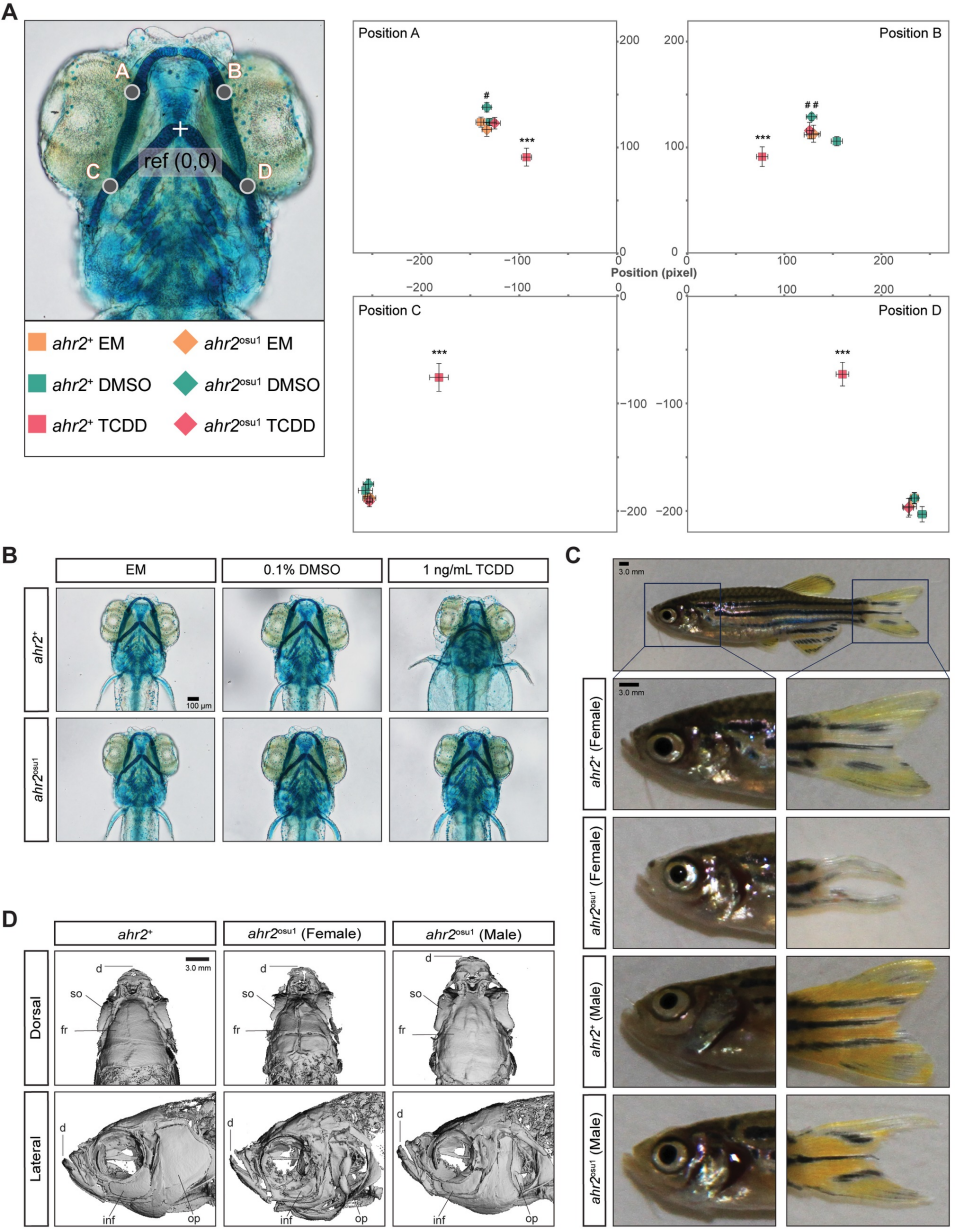
Fin and skeletal abnormalities observed in adult *ahr2*^osu1^ line. Wild types and AHR2-null zebrafish were developmentally exposed at 6 hpf to EM, 0.1% DMSO, or 1 ng/mL TCDD, and the cartilage was stained and measured at 5 dpf. **(A)** A morphometric system was used to measure the position and length of landmark structures in the developing jaw. The position of jaw structures representing junctions between Meckel’s and palatoquadrate cartilages (points A and B) and the hyosymplectic and ceratohyal cartilages (points C and D) was measured relative to a reference point as shown. Statistical significance was determined by a modified two-way ANOVA with a Tukey *post hoc* test. Morphometric values represent mean ± SD (*n* = 9–10; *p* < 0.05 = * or #, *p* < 0.01 = ** or ##, *p* < 0.001 = ***). The asterisk (*) indicates statistical significance in wild-type fish exposed to TCDD compared to wild-type fish exposed to DMSO, and (#) indicates statistical significance in DMSO-exposed *ahr2*^osu1^ mutants compared to DMSO-exposed wild-type fish. **(B)** Representative ventral views of 5 dpf cartilage in wild types and AHR2-null mutants developmentally exposed to EM, 0.1% DMSO, or 1 ng/mL TCDD. Black bar in bottom right corner = 100 μm. **(C)** Representative brightfield images of adult wild types’ and *ahr2*^osu1^ mutants’ heads and caudal fins. Dark blue square represents area in panels below. Black bar in top left corner = 3.0 mm. **(D)** microCT imaging of adult wild-type (female) and *ahr2*^osu1^ mutant female and male zebrafish heads. Male and female wild-type microCT scans looked the same and a representative female image was selected as the representative image. Notable differences were observed in the dentate (d), supraorbital (so), frontal (fr), infraorbital (inf), and operculum (op). Black bar in top right corner = 3.0 mm.

TCDD-activation of the AHR2 pathway during development produces craniofacial cartilage defects resulting in smaller and abnormal cartilage structures [28]. It is unclear whether the AHR2 regulates cartilage development in the absence of an exogenous ligand, such as TCDD. To determine if the AHR2 is required for proper development of craniofacial cartilage, we stained and measured the cartilage of 5 dpf zebrafish. A morphometric system was used to measure the position and length of landmark structures in the developing jaw as described in [28]. The cartilage of *ahr2*^+^ and *ahr2*^osu1^ larvae was not significantly different, suggesting the AHR2 is not required for early development of the craniofacial cartilage in 5 dpf zebrafish (Fig 5A and 5B**)**. To further asses the function of the AHR2 in our mutant line, we developmentally exposed *ahr2*^+^ and *ahr2*^osu1^ zebrafish to 0.1% DMSO or 1 ng/mL TCDD for 1 hour at the shield stage (~6 hpf) and measured the relative position of the cartilage at 5 dpf. As expected, the wild-type line exhibited significant malformations in the craniofacial cartilage structures, while the mutant line was resistant to TCDD-induced malformations (Fig 5A and 5B). Unexpectedly, a comparison between the DMSO-exposed *ahr2*^+^ and *ahr2*^osu1^ zebrafish resulted in a significant difference in the relative position of Meckel’s and palatoquadrate cartilages (points in Fig 5A and 5B).

We have previously reported that *ahr2*^hu3335^ mutants generated by the TILLING method have skeletal and fin defects in adulthood [20]. To determine if our CRISPR-generated line recapitulated this phenotype, we imaged adult zebrafish (*n* = 4 male and 4 female per genotype) and qualitatively examined the fins. All *ahr2*^osu1^ zebrafish examined displayed defective dorsal, ventral, and caudal fins; however, the females produced a more severe phenotype compared to the males (Fig 5C). The *ahr2*^osu1^ zebrafish line also had visible jaw malformations compared to the wild type.

To examine the head skeleton in a non-destructive manner, we used microCT to scan adult zebrafish (*n* = 2 male and 2 female per genotype). MicroCT imaging revealed structural differences between the craniofacial skeleton of *ahr2*^+^ and the *ahr2*^osu1^ lines, with the female *ahr2*^osu1/+^ mutants displaying more severe skeletal defects than their male counterparts (Fig 5D). The notable differences in skeletal anatomy included abnormal dentary, operculum, and frontal structures, and smaller orbital and supraorbital bones. These results are concordant with the phenotype of the TILLING-generated *ahr2*^hu3335^ mutant line, confirming that AHR2 is required for proper adult skeleton formation in zebrafish.

### Zebrafish AHR2 is required for normal larval and adult behavioral responses

**Fig 6:**
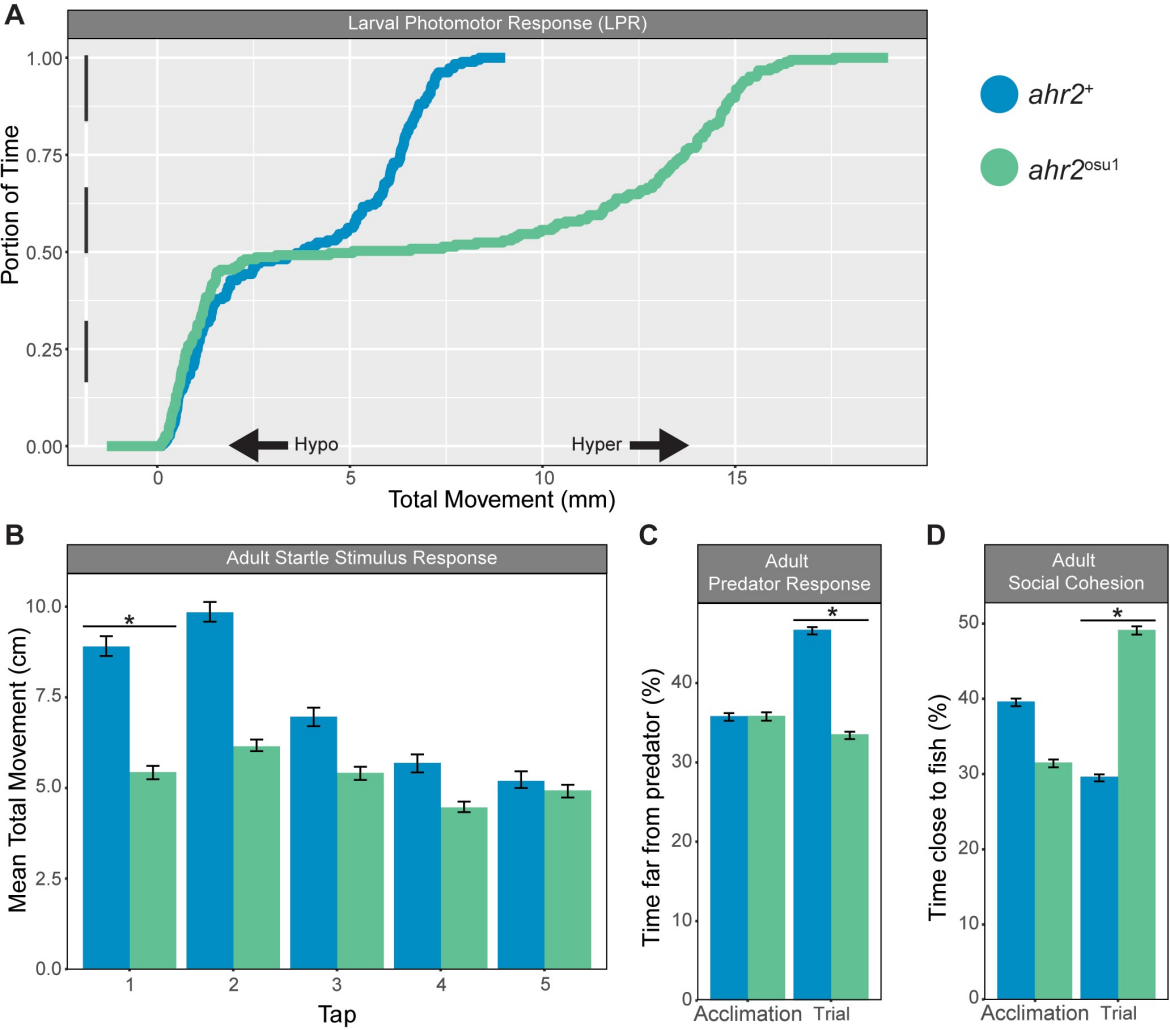
Larval and adult behavior irregularities observed in adult *ahr2*^osu1^ mutant zebrafish. **(A)** Larval photomotor response (LPR) in wild types and *ahr2*^osu1^ mutants at 5 dpf (*n* = 32) using the ViewPoint ZebraBox systems. The LPR assay consisted of 3 minutes of light and dark alternating periods, for four light-dark transitions, with the first transition representing an acclimation period. The black and white bar along the y-axis indicates the 3 minutes of light (white) and dark (black) alternating periods. Larval zebrafish at this developmental stage display increased locomotion during periods of darkness. The overall area under the curve was analyzed for the last 3 light-dark cycles compared to control morphants using a Kolmogorov-Smirnov test (*p* < 0.01). **(B-D)** Wild-type and *ahr2*^osu1^ mutant adult behavioral response including startle stimulus **(B)**, predator response (C), and social cohesion (D) assays. Statistical differences between mutants and controls were determined by two-way ANOVA with repeated measures and Tukey HSD *post hoc* test (*p* < 0.05).

To evaluate the functional role of AHR2 on larval locomotor behavioral responses, 120 hpf fish (*n* = 32) were subjected to a light-dark larval photomotor response assay (LPR) that determines hyperactive (increase) or hypoactive (decrease) swimming activity in response to a light stimulus as described in (Knecht et al., 2016). The *ahr2*^osu1^ line displayed a significant hyperactive locomotor response (*p* < 0.01; 91.6% change in mean area under the curve) in the light period compared to wild type (Fig 6A). The LPR assay suggests that the absence of a functional AHR2 protein significantly affected some aspect(s) of neuromuscular and/or sensory system development.

We also examined the functional role of AHR2 in adult zebrafish behavior. To evaluate anxiety related behaviors, we used the startle response and predator avoidance assays as described in [38]. The *ahr2*^+^ fish produced the expected startle response, consisting of an initial increase in movement following the first two taps, followed by habituation to the tap stimulus. The *ahr2*^+^ fish displayed a significant increase in the mean total movement in response to the first tap compared to the AHR2-null mutants (Fig 6B). The *ahr2*^osu1^ fish did not increase their movement following any of the taps. In the predator response assay, the fish are expected to flee from a video clip of a predator fish consuming a zebrafish. The *ahr2*^+^ zebrafish produced the expected predator avoidance behavior, while the *ahr2*^osu1^ fish did not appear to respond to the predator stimulus (Fig 6C). To evaluate social behaviors, the social cohesion assay measures the percent of time the fish spend close to an LCD video projection of a school of free-swimming zebrafish. The *ahr2*^osu1^ fish spent a significantly greater percent of time near the zebrafish stimulus (LCD display of zebrafish) compared to the *ahr2*^+^ fish (Fig 6D). In summary, the larval and adult behavior assays demonstrate that AHR2 is required for the development and/or adult homeostasis of neuromuscular and/or sensory systems.

## Discussion

The aryl hydrocarbon receptor functions as an environmental sensor connecting extracellular environmental signals to internal cellular processes, allowing the cell to rapidly respond to both endogenous and exogenous ligands. The AHR is necessary for proper vertebrate development and homeostasis, while activation of AHR by ubiquitous environmental pollutants can lead to adverse developmental and cognitive effects in wildlife and humans [11, 12]. Disruption of AHR signaling is associated with several diseases, including prostate and coronary artery disease [11, 42, 43]. Understanding the AHRs fundamental roles in biology may foster a better understanding of the target organs and molecular mechanisms by which inappropriate AHR activation leads to toxicity.

In this study, we used the CRISPR-Cas9 system to generate a zebrafish mutant line (*ahr2*^osu1^) to improve and expand upon the AHR2-null phenotypes reported in our previous publication [20]. The Goodale report has two limitations that make it difficult to definitively link observed phenotypes to a mutation in AHR2: 1) TILLING produces a high degree of non-specific background mutations, and 2) the point mutation produced by the TILLING method occurs in the transactivation domain, which leaves the possibility of the protein retaining some biological function since the bHLH (DNA binding) and PAS (ligand binding) domains were unaffected. The CRISPR-generated mutant is predicted to produce a truncated protein with a premature stop codon upstream of the nuclear localization and export signals, and the bHLH, PAS, and transactivation domains. A majority of the phenotypes examined were recapitulated or approximated in the TILLING-generated ahr2^hu3335^ mutant line, providing additional evidence that the observed phenotypes were due to an *ahr2* mutation. AHR2 is required to produce TCDD-induced toxicity in zebrafish [44]. We used this well-characterized response to determine whether the CRISPR-generated mutant line has a functional AHR2 protein. The *ahr2*^osu1^ line was TCDD-resistant, supporting the hypothesis that AHR2 is not functional and, being a CRISPR line, is a likely much cleaner background for investigating the biological and toxicological functions of AHR2. The advantage of the new *ahr2*^osu1^ line is that it is a second line to confirm the AHR2 genotype phenotype connection and it produces a (predicted) truncated protein without any functional domains.

In response to TCDD exposure, expression of cytochrome p450 *cyp3a65* and *slincR* transcripts were significantly increased in the wild type line. AHR2 is required for the TCDD-induced increase of *cyp3a65* and *slincR* transcripts, providing further support for the hypothesis that AHR2 is not functional in the new *ahr2*^*osu1*^ line [40, 41]. The expression of cytochrome p450s (*cyp1a*, *cyp1b*, *cyp1c1*), *ahhra*, and *ahrrb* transcripts increased in both the TCDD-exposed wild type and mutant lines at 5 dpf when compared to the vehicle control; however, the wild type line had a significantly greater increase in expression compared to the AHR2-null line. The increase in expression in the mutant line is most likely due to the AHR1B isoform, which can bind to TCDD with high affinity [45]. The TILLING-generated *ahr2*^*hu3335*^ line evaluated the expression of *cyp1a*, *cyp1b1*, *cyp1c1*, and *cyp1c2* in response to TCDD exposure at 48 hpf. None of the transcripts had a significant increase in expression in the *ahr2*^*hu3335*^ mutant line, suggesting the 48 hpf time point may be more appropriate for determining ARH2 functionality and identifying AHR2 specific ligands. It should be noted that the TCDD-induced developmental toxicities observed in zebrafish are a Cyp1a-independent mechanism (45). This explains why the new *ahr2*^*osu1*^ line is resistant to TCDD-induced toxicities in the presence of an increase in expression of the cyp genes

The previously characterized AHR2-null line reported impaired spawning activity of homozygous crosses [20]. In concordance with the TILLING line, the CRISPR-generated AHR2-null line exhibited reduced fecundity, abnormal oocyte development, and histological evaluations indicated an increase in egg associated inflammation. At 36 weeks, the *ahr2*^osu1^ line had a significant decrease in mature follicles (stage IV) and a significant increase in atretic follicles suggesting that AHR2 plays a role in normal ovarian follicle growth and development. This finding correlated with an increased percentage of mid-vitellogenic (stage III) follicles, suggesting an inhibition of vitellogenin-dependent stages of growth. Similarly, in AHR knockout mice, histological evaluation of adult ovaries exhibited a reduction in the number of mature follicles when compared to wild type ovaries [46]. In mice, AHR is hypothesized to regulate follicle growth through impaired estradiol production and reduced gonadotropin responsiveness and that the role of AHR is dependent upon the stage of sexual maturity [47, 48]. In mammalian female reproductive functions, the AHR signaling pathway may also regulate fertility, embryo nourishment, maintenance of pregnancy, and normal ovarian function [47]. Furthermore, the ectopic activation of AHR by exogenous ligands (2,3,7,8-TCDD) elicits similar impacts on follicular development in zebrafish [49] as well as rodents [50]. Further experiments are required to determine the full role of normal and ectopic AHR2 signaling in zebrafish female reproductive system development and function.

In zebrafish and mammalian models, developmental exposure to TCDD results in abnormal cranial cartilage development [51, 52]. In zebrafish, repression of the transcription factor *sox9b* is hypothesized to have a causal role in TCDD-induced craniofacial cartilage malformations [28]. At 48 hpf, the repression of *sox9b* is mediated by the AHR2-dependent increase in expression of a long non-coding RNA called *slincR* that binds to the 5’ untranslated region of the sox9b promoter to repress transcription ([40] and Garcia and Tanguay (manuscript in preparation). To determine if AHR2 has a functional role in normal cartilage development, we stained and measured the cartilage of 5 dpf zebrafish. The cartilage of *ahr2*^osu1^ fish developed normally; however, exposure to 0.1% DSMO resulted in a significant difference in the relative position of Meckel’s and palatoquadrate cartilages when compared to the wild type line. Previous reports have shown that zebrafish exposed to 0.1% DMSO exhibit signs of toxicity, including an induction of stress proteins and a decrease in heart rate [53]. Our results suggest that the absence of a functional AHR2 signaling pathway and exposure to 0.1% DMSO results in subtle malformations in the cartilage structure. We lack sufficient data to make any conclusion regarding the functional role of AHR2 in DMSO exposed embryos. In the absence and presence of TCDD, the *ahr2*^osu1^ fish did not have a significant difference in expression of *sox9b* or *slincR* when compared to controls; however, the wild-type fish had a significant increase in *slincR* and decrease in *sox9b* expression when exposed to TCDD. It appears that AHR2 is not required for proper zebrafish cartilage development; conversely, the inappropriate activation of AHR2 results in impaired cartilage development.

The *ahr2*^hu3335^ line is associated with abnormal skeletal bone structures and damaged fins in adults [20]. The CRISPR-generated *ahr2*^osu1^ line recapitulated the previously reported defective skeletal and fin phenotypes. These results indicate that AHR2 is required for the proper formation or maintenance of the cranium and fins in adult zebrafish. Additionally, our data suggest that AHR2-null females may have more severe loss-of-function skeletal and fin phenotypes when compared to AHR2^-/-^ males; however, further experiments are required, since we only observed a limited number of fish in a sex specific manner.

Our study evaluated the functional role of AHR2 on neurological and locomotor behavior in larval and adult zebrafish. The data suggest that the absence of a functional AHR2 protein significantly affected some aspect(s) of neuromuscular and/or sensory system development, with impaired behavioral responses observed into adulthood. We cannot rule out that the altered behavioral responses in the AHR2-null line was not due to subtle skeletal or fin malformation and thus more of mechanical effect on swimming efficiency; however, evidence from other model organisms suggest AHR regulates the development of neural and sensory systems. In the nematode (*Caenorhabditis elegans*), loss-of-function studies suggest that AHR-1 (homolog of AHR) regulates neuronal cell differentiation, migration, and cell fate determination, with a functional role beyond embryonic neural development [54]. The AHR homolog in the fruit fly (*Drosophila melanogaster*), spineless, is required for proper development of distil segments of antennae (multi-sensory structures), specifying photoreceptor cell fate of the compound eye, and controlling dendrite morphology and branching in sensory neurons of the peripheral nervous system [55–57]. In the developing mouse, overexpression of AHR also results in abnormal dendrite morphology and position of cortical neurons [58]. AHR-null mice and TCDD-exposed mice both displayed reduced neuronal cell differentiation, fewer mature neurons, and impaired cognitive responses [59, 60]. In zebrafish, developmental exposure to the AHR2 ligand benzo[*a*]pyrene, results in the transgenerational inheritance of abnormal neurobehavioral responses observed in larval and adult zebrafish [38]. Zebrafish developmentally exposed to 1,9-benz-10-anthrone had morphological malformations and a decrease in the expression of genes involved in sensory and visual perception (in an AHR2-dependent mechanism) [61]. The results from our research are in concordance with previously published data and supports the hypothesis that one of the ancestral roles of AHR involves regulating the development and function of sensory structures and neural systems [1].

In summary, we used the CRISPR-Cas9 system to generate an AHR2-null line that will aid in investigating AHR’s functional roles in biology and toxicology. In zebrafish, the absence of a functional AHR2 protein results in decreased survival and negatively impacted female reproductive health. AHR2-null zebrafish display abnormal skeletal bone structures and severely damaged fins. Our data suggest AHR2 is required for the proper development of some aspect(s) of the neuromuscular and/or sensory system, with impaired behavioral responses observed in larval and adult zebrafish. This report increases our knowledge of the role of AHR in organ development and homeostasis, which should lead to a better understanding of the target organs and molecular mechanisms involved in AHR-mediated toxicities.

## Supporting information

S1 Table List of all primers and oligos. (DOCX)

S1 File ImageJ customized set origin tool macro. (TXT)

S2 File ImageJ customized click coordinates tool macro. (TXT)
