# Supplementary material for: AHR2 required for normal behavioral responses and proper development of the skeletal and reproductive systems in zebrafish
Source: PLoS One. 2018 Mar 1;13(3):e0193484. doi: 10.1371/journal.pone.0193484 (PMC5832240; doi:10.1371/journal.pone.0193484)
Supplement: Supplementary file 1 [file pone.0193484.s001.docx]

Supplemental Table 1 List of all primers and oligos

Application	Gene	(5'-3')	
sgRNA assembly	AHR2 sgRNA 1 oligo	GCGATTTAGGTGACACTATA**GGCGGGTATCGGTACATATG**GTTTTAGAGCTAGAAATAGC	
	AHR2 sgRNA 2 oligo	GCGATTTAGGTGACACTATA**GACATATGCGGTCAAGAAA**GTTTTAGAGCTAGAAATAGC	
	Constant (generic) oligo (Sp6 promoter)	AAAAGCACCGACTCGGTGCCACTTTTTCAAGTTGATAACGGACTAGCCTTATTTTAACTTGCTATTTCTAGCTCTAAAAC	
	Universal Forward	AAAGCACCGACTCGGTGCCAC	
	Universal Reverse	GCGTAATACGACTCACTATAG	
		Forward (5'-3')	Reverse primer (5'-3')
Melt Curve analysis	AHR2 sgRNA 1	ATACCAGACAACCATGTCGG	TTTCTGAACGGGCTTCTTCC
Sequencing Primers	AHR2	TTCAACAGTCCTCCTTAAGAACG	TGTAAAATAACAACATAACTTGGCCC
qRT-PCR	β-actin	AAGCAGGAGTACGATGAGTC	TGGAGTCCTCAGATGCATTG
	AHR2	TTCAACAGTCCTCCTTAAGAACG	TGTAAAATAACAACATAACTTGGCCC
	ahrra	CCCTGAAGTGTGTGTTGAACAGAG	TGCTGCATGTACCCTCTGTAGTC
	ahrrb	CTGAATGCAGAGCTGGACCG	AAGCTGACGCTGAGTCTCAGC
	cyp1a	TGCCGATTTCATCCCTTTCC	AGAGCCGTGCTGATAGTGTC
	cyp1b1	TCATCCTGCTACTTGTCAGG	TGGATGTGTCTTTGGTCGTG
	cyp1c1	AGTGGCACAGTCTACTTTGAGAG	TCGTCCATCAGCACTCAG
	slincR	GATTACACTCACCCCGCAGC	CAATCCTCAGTGTCCGTCAGT
	sox9b	GGAGAAGTTCCCGGTGTGTA	GCTTGAGCCCAAACCATAAA
	wfikkn1	CTGTCCAGCCCAAACTCC	TCTGGGTGTCTGCGTCTGTC
